# The Influence of Surface Preparation, Chewing Simulation, and Thermal Cycling on the Phase Composition of Dental Zirconia

**DOI:** 10.3390/ma14092133

**Published:** 2021-04-22

**Authors:** Markus Wertz, Florian Fuchs, Hieronymus Hoelzig, Julia Maria Wertz, Gert Kloess, Sebastian Hahnel, Martin Rosentritt, Andreas Koenig

**Affiliations:** 1 Department of Prosthodontics and Material Sciences, Leipzig University, 04103 Leipzig, Germany; Florian.Fuchs@medizin.uni-leipzig.de (F.F.); sebastian.hahnel@medizin.uni-leipzig.de (S.H.); 2 Institute of Mineralogy, Crystallography and Materials Science, Leipzig University, 04103 Leipzig, Germany; hieronymus.hoelzig@uni-leipzig.de (H.H.); kloess@uni-leipzig.de (G.K.); 3 Institute of Communication Science, University of Hohenheim, 70599 Hohenheim, Germany; Julia.wertz@uni-hohenheim.de; 4 Department of Prosthetic Dentistry, Regensburg University Medical Center, 93053 Regensburg, Germany; Martin.rosentritt@ukr.de

**Keywords:** yttria-stabilized zirconia, X-ray diffraction, Rietveld, roughness, dental restauration, monoclinic, tetragonal

## Abstract

The effect of dental technical tools on the phase composition and roughness of 3/4/5 yttria-stabilized tetragonal zirconia polycrystalline (3y-/4y-/5y-TZP) for application in prosthetic dentistry was investigated. Additionally, the X-ray diffraction methods of Garvie-Nicholson and Rietveld were compared in a dental restoration context. Seven plates from two manufacturers, each fabricated from commercially available zirconia (3/4/5 mol%) for application as dental restorative material, were stressed by different dental technical tools used for grinding and polishing, as well as by chewing simulation and thermocycling. All specimens were examined via laser microscopy (surface roughness) and X-ray diffraction (DIN EN ISO 13356 and the Rietveld method). As a result, the monoclinic phase fraction was halved by grinding for the 3y-TZP and transformed entirely into one of the tetragonal phases by polishing/chewing for all specimens. The tetragonal phase t is preferred for an yttria content of 3 mol% and phase t″ for 5 mol%. Mechanical stress, such as polishing or grinding, does not trigger low-temperature degradation (LTD), but it fosters a phase transformation from monoclinic to tetragonal under certain conditions. This may increase the translucency and deteriorate the mechanical properties to some extent.

## 1. Introduction

Over the past 20 years, yttria-stabilized tetragonal zirconia polycrystalline (Y-TZP) has become increasingly popular for application in dental restorations [1,2,3].

Compared to other dental materials, Y-TZP has an outstanding mechanical performance [4] (flexural strength between 750 and 1300 MPa [5,6]). The material is biologically inert [6] and has a high X-ray opacity, and, depending on the yttria content, its optical properties such as translucency [7] are similar to those of enamel.

The materials are pre-sintered industrially and can be easily processed automatically in blocks and blanks using computer aided manufacturing (CAM) [8]. Y-TZP ceramics may be applied in monolithic dental restorations, such as fixed dental prostheses (FDPs) and abutments [5,8,9].

A disadvantage of Y-TZP ceramics is its low-temperature degradation (LTD). This aging phenomenon is characterized by slow tetragonal (t) to monoclinic (m) phase transformation [10,11,12,13], is caused by thermal [3,14,15] or mechanical [2,16,17] stress, and is fostered by humidity [14,16].

It may impair mechanical and physical properties of the material and lead to the formation of micro cracks at the grain boundaries and even to complete failure of restoration [14,16,18]. Typical examples include the catastrophic failure of more than 200 femoral heads of hip implants that occurred between 2000 and 2002 [19] as well as the failure of 13 of 200 dental implants in a batch from 2003 to 2007 [20]. In dentistry, meta-analysis shows that the overall survival rate of one- and two-piece dental implants fabricated from zirconia can be calculated to be 92% after one year of clinical service [21].

Y-TZP features several crystallographic modifications: as a mineral and under standard conditions, ZrO_2_ is monoclinic (P2_1_/c); under high-temperature conditions, there are three tetragonal (P4_2_/nmc) phases and one cubic (Fm$\bar{3}$m) phase [22,23,24,25,26].

The cubic phase features a fluorite structure type [27] and transforms into a tetragonal (t, t′, t″) phase with decreasing yttria content and temperature [22,23,24].

The three tetragonal phases differ in volume and result from phase separation caused by quenching of Y-TZP after sintering. According to Lipkin et al. [24], the yttria-lean phase components initially transform to the tetragonal phase t, which is followed by a transformation to the monoclinic phase. The yttria-rich phase components transform to the tetragonal phase t″ and the cubic phase c [24].

The tetragonal phases are slightly distorted and connected with the cubic phase by a clear group-subgroup chain. Thus, the transformation from a cubic to a tetragonal phase is not disruptive [22,23].

However, this circumstance does not apply to the phase transformation from the tetragonal phase t to the monoclinic phase m. This process coincides with a substantial volume change and a large shear strain of from 8% [28] to 9% [21]. This may lead to micro cracking, twinning [28], and an increasing amount of surface irregularities [18]. The consequences include an increasing loss of flexural strength [29] and, in the worst case, failure [19,20] associated with the increasing proportion of the monoclinic phase [14,16,18,24].

The phase transformation from the tetragonal to the monoclinic phase (m → t) may initially increase the fracture toughness. However, with a further increase in the monoclinic phase, this effect vanishes [28]. It has been postulated [24] that only the tetragonal phase t may transform into the monoclinic phase [24], so it is important to also examine the other phases.

In contrast to these considerations, Bothelo et al. [29] postulated that mechanical processing (e.g., grinding and polishing) does not influence the monoclinic phase fraction, suggesting that a monoclinic phase fraction in 3y-TZP (Cercon base from Dentsply Sirona) does not exist, despite the fact that processing and mechanic processing partly restore some mechanical properties, such as flexural strength.

The current study aims to investigate the influence of different dental technical tools employed for the fabrication and processing of zirconia restorations as well as aging processes, such as thermocycling (TC) and mechanical loading on the phase composition (m, t, t′, t″, c) of zirconia with three different yttria contents.

The null hypothesis is that the monoclinic phase fraction and the roughness (Sa) will increase with increasing mechanical (grinding, polishing, and chewing) and hydrothermal (thermocycling) stress.

## 2. Materials and Methods

### 2.1. Materials

The seven types of commercially available zirconia that can be applied to the fabrication of dental restorations plates were fabricated using a dental furnace (inFire HTCspeed sintering furnace, Dentsply Sirona, Bensheim, Germany; program: Pre-heating with 10 K/min up to 1450 °C; holding time: 120 min; cooling off: 10 K/min) and a five-axis milling machine (Sirona inLab MC XL, Dentsply Sirona, Bensheim, Germany). For polishing, a Dental Direkt Panther edition Kit (Dental Direct GmbH, Spenge, Germany, Lot #61525) was used. Treatment included 6 min with a “Panther Flame 055M at 15,000 U/min”, 5 min with a “Panther Flame 055XF at 15,000 U/min”, and 2 min with a goat hair bur (19 mm) and Panther diamond polishing agent (Table 1).

**Figure 1:**
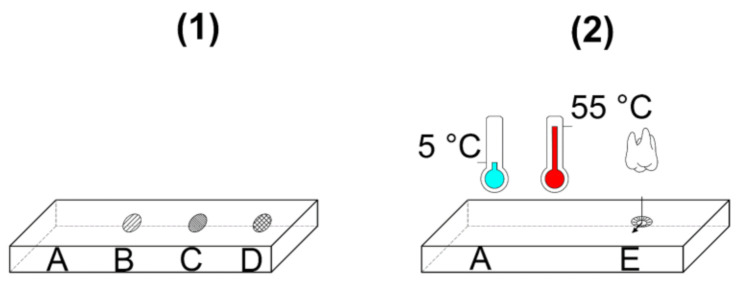
Visualization of the measurement points on the surface of two zirconia specimens for each treatment code (abbreviations in Table 2). (**1**): without thermocycling (TC); (**2**) with thermocycling (TC) and chewing simulation.

The plates were subjected to three mechanical treatments and one thermal treatment, resulting in an overall total of six measurement positions (Table 2/Figure 1).

Figure 1 displays the different treatments applied on the surface of each specimen. Due to the thermal cycling, we distributed the measurement points (treatments) over two different plates.

### 2.2. Methods

#### 2.2.1. Confocal Laser Scanning Microscopy (CLSM)

A Keyence VK-X1000/1050 (Keyence Deutschland GmbH, Neu-Isenburg, Germany) confocal laser scanning microscopy (CLSM) with a Nikon CF IC EPI Plan 50X (NA: 0.5, NIKON, Osaka, Japan) objective was used for surface measurement. Sa as the amount of the height difference of each point compared to the arithmetic mean of the surface was evaluated with Multi File Analyzer software (2.1.3.89, Keyence Deutschland GmbH, Neu-Isenburg, Germany) according to ISO 25178-2:2012. Therefore, five different areas per samples were characterized five times each with an area of 100 µm × 100 µm each and filtered to receive the surface for roughness evaluation (S-Filter: 0.5 µm; F-Filter: 0.1 mm; Filter type: spline).

As values identified for Sa were not normally distributed across the samples of all materials analyzed in this study (Shapiro-Wilk test [30]: *p* < 0.05), medians and 25/75 percentiles were calculated, and statistical analyses were performed using the Kruskal–Wallis test [31] and post hoc analyses using the Dunn [32] post hoc test with Bonferroni [33] correction. To determine the strength of the effects, Cohen’s r [34,35] was calculated using the psychometrica platform. The general level of significance (*α*) was set to 0.05, and for interpretation, the Bonferroni-adjusted *p*-values were used.

#### 2.2.2. X-ray Diffraction (XRD)

All X-ray diffraction (XRD) measurements were performed using a Bruker D8 discover (Bruker AXS Advanced X-ray Solutions GmbH, Karlsruhe, Germany) with CuKα radiation (1.54 Å, 40 mA, 40 kV).

The measuring spot was between 1 and 4 mm^2^ in size, and the snout diameter was 0.5 mm.

A VÅNTEC-500 (Vantec Thermal Technologies, Fremont, CA, USA) was used as area detector, and three frames (1200 s each) from 25° to 75° with a 15° interval between the frames were measured. The goniometer radius on the secondary side was 300 mm.

Using a measurement spot between 1 and 4 mm^2^ in size, a large surface area was measured, which produced robust data in each single measurement due to the small crystallite size of the material and, hence, the high number of measured grains.

The diffractograms were integrated with the program DIFFRAC.EVA (Version 3.1; Bruker AXS Advanced X-ray Solutions GmbH, Karlsruhe, Germany); indexing of the registered reflexes was performed by means of structure maps from the ICDD-PDF2 database as well as [36].

Two different principles of quantitative phase analysis were applied as follows:

(a) Semi quantitative phase analyses (XRD).

The proportion of the monoclinic phase was quantified according to DIN EN ISO 13,356 [37] on a polished surface using the equation of Garvie and Nicholson [38].

(1) $$\alpha =\frac{M\left(\bar{1}11\right)+M\left(111\right)}{M\left(\bar{1}11\right)+T\left(111\right)+M\left(111\right)}$$

*α* is the proportion of the monoclinic phase, *M*(hkl) denotes the intensity of monoclinic reflections of different indexing, and *T* (111) is the intensity of a tetragonal reflection. Prior to the calculations, the background was subtracted to avoid relevant overestimation of the monoclinic fraction.

The volume fraction of the monoclinic phase was determined according to Toraya et al. [39].

(2) $$V_{m}=\frac{1.311\propto }{1+0.311\propto }$$

The results of less than one percent were assumed not to have a monoclinic phase in the sample.

(b) Rietveld refinement.

Rietveld refinement was performed using the software TOPAS (Version 4.2, Bruker AXS Advanced X-ray Solutions GmbH, Karlsruhe, Germany).

While ICSD (Inorganic Crystal Structure Database; Karlsruhe, Germany, https://icsd.fiz-karlsruhe.de/index.xhtml, accessed on 22 November 2021) structural data are available for the phases m, t, and c, the structural models for phases t′ and t″ were developed using literature data [13,25,36] and included in the Rietveld refinement.

**Figure 2:**
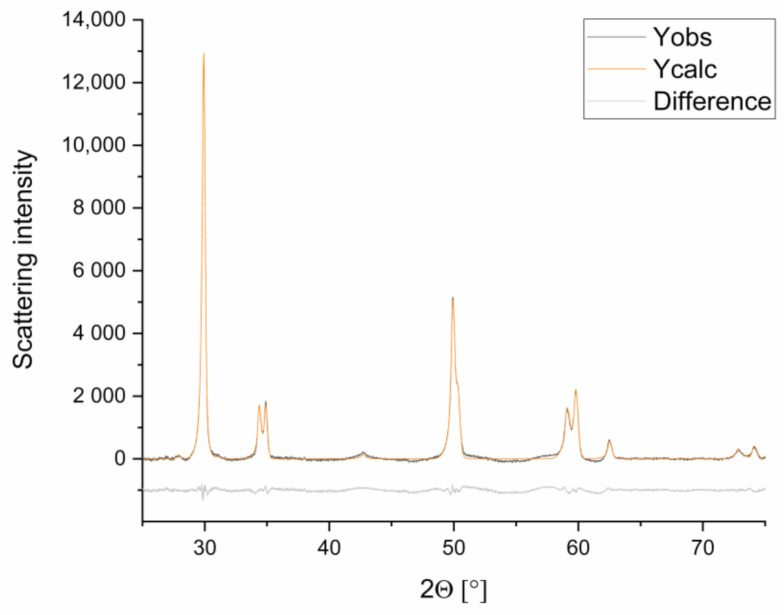
Comparison of a refined (Ycalc) and a measured (Yobs) graph, and the difference between the two graphs (used sample: 3y_PM (1)B), Rwp: 1.5.

The crystallite size, the lattice constants, the site occupancy factor (S.O.F.) of the individual atom positions, and the isotropic temperature form were refined. The refinements achieved by this procedure fitted the measured diffractogram. The Rwp values (parameter for assessing the quality of the refinement, weighted profile R-value) ranged between 1.5 and 2.2 (example displayed in Figure 2).

**Figure 3:**
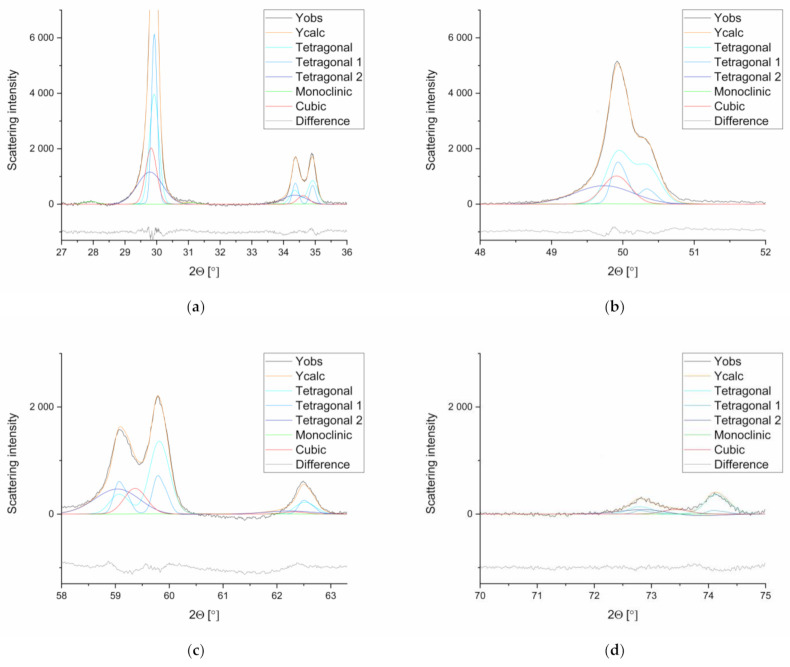
Comparison of the different calculated phases, the observed graph (Yobs), the refined graph (Ycalc), and the difference between Yobs and Ycalc (used sample: 3y_PM (1)B). (**a**) 27–36°, (**b**) 48–52°, (**c**) 58–64°, (**d**) 70–75°.

The pronounced overlapping of reflexes can be explained by the strong similarity of the tetragonal and cubic phases. Notably, between the phases t and t′ as well as t″ and c, the transition is fluent (cf. Figure 3) [22,24], which becomes clear by comparing the “tetragonality” (a_pseudocubic_ = a × 2^0.5^) of the tetragonal phases [36] (t: 1.0159, t′: 1.0149, t″: 1.0033). Only at high diffraction angles (particularly around 59° and 73°) were reflexes (visualized as peaks in the diffractogram) identified, which allowed a distinction.

The consequence of this flowing transition between the tetragonal phases is that overlaps occur between the tetragonal phases t and t′ and between the phases t′′ and c, so portions of one phase can be misinterpreted for the other.

## 3. Results

### 3.1. Surface Roughness

**Figure 4:**
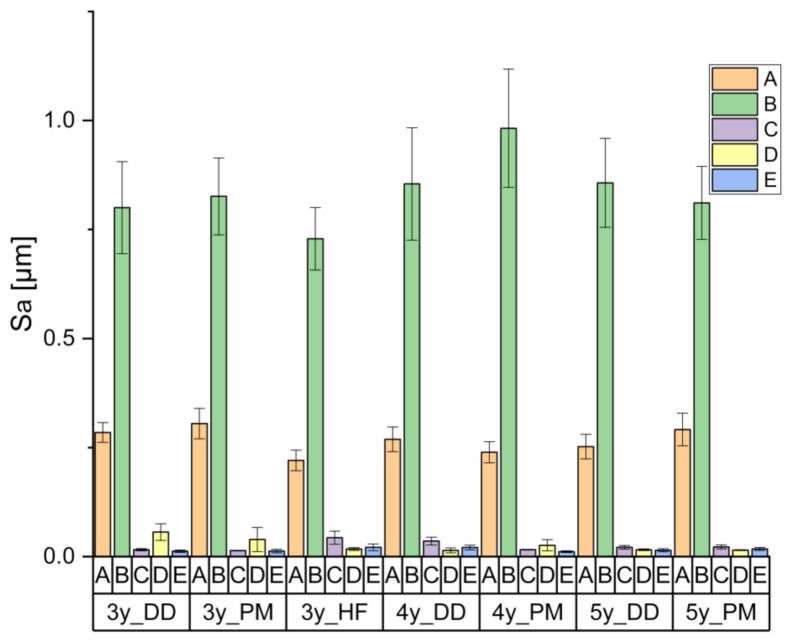
Comparison of Sa between the different Y-TZPs (Table 1) and treatments (Table 2: A—untreated, B—grinding, C—grinding and polishing, D—polishing and E—grinding, polishing, chewing, and thermocycling).

Sa increased significantly after grinding (B) and decreased after polishing (C + D). Chewing simulation (E) did not significantly influence Sa (Figure 4).

The Kruskal–Wallis test indicated significant differences in Sa between the various ceramics.

Sa in measurement position A (untreated) did not differ significantly from B (grinding) but significantly differed from the positions C (grinding and polishing), D (polishing), and E (grinding, polishing, chewing, and thermocycling). Position B significantly differed on the samples of all the studied materials from all groups except group A.

No significant differences in Sa were identified in the positions C (grinding and polishing), D (polishing), and E (grinding, polishing, chewing, and thermocycling) between the various Y-TZP ceramics.

Detailed information regarding the results of the statistical analyses can be retrieved in the Appendix (Table A1, Table A2, Table A3, Table A4, Table A5, Table A6 and Table A7).

### 3.2. X-ray Diffraction (XRD)

(a) Semi quantitative phase analyses (XRD)

**Figure 5:**
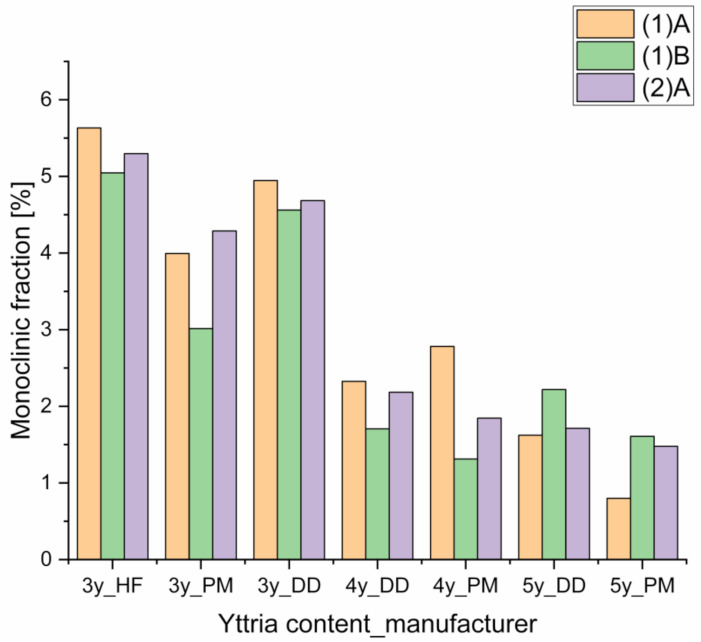
Monoclinic phase fraction of the different Y-TZP ceramics with different yttria contents (3–5 mol%) and different manufacturers identified at positions (1)A (untreated), (2)A (after thermocycling), and (1)B (after grinding) (cf. Figure 1) determined according to the formula of Nicholson and Garvie [38,39].

X-ray data evaluated using the formula of Garvie and Nicholson [38] and the correction by Toraya [39] showed a monoclinic phase fraction that was dependent on the yttria content. No dependency on the type of processing was observed (cf. Figure 5).

Polishing and chewing simulation led to the disappearance of the monoclinic phase fraction. However, a halving of the proportion of the monoclinic phase from processing step (1)A to processing step (1)B is not recognizable with this method (cf. Figure 5).

(b) Rietveld refinement

**Figure 6:**
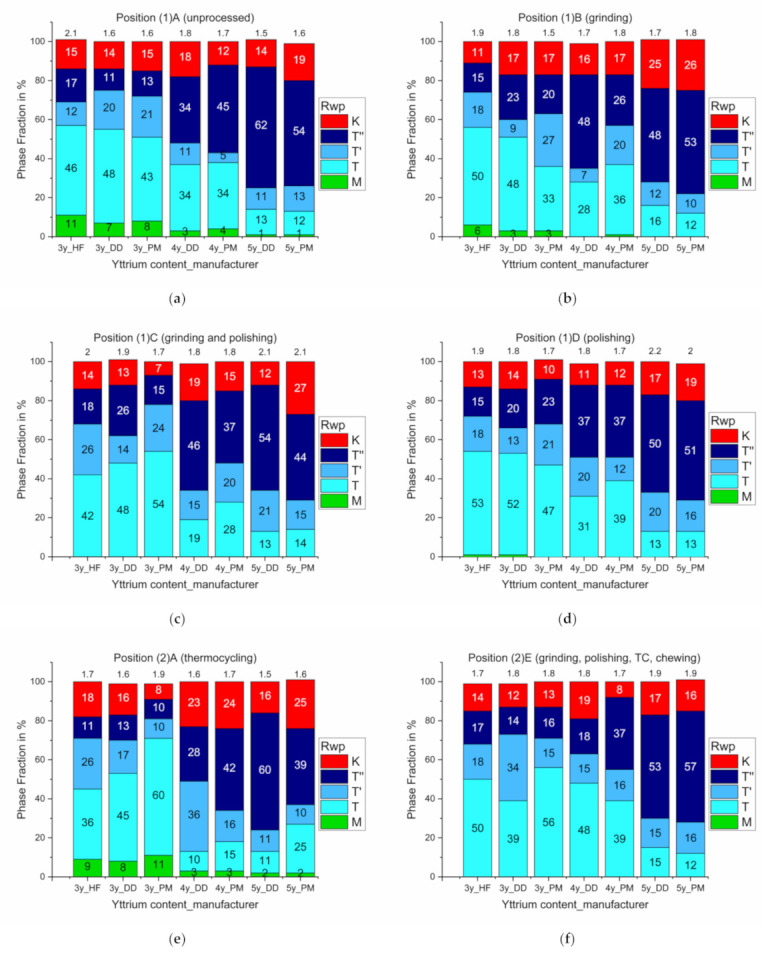
Comparison of six different measurement positions with their individual phase fractions and Rwp values: (**a**): baseline, (**b**): grinding, (**c**): grinding and polishing, (**d**): polishing, (**e**): thermocycling, (**f**): grinding, polishing, thermocycling, and chewing).

The properties of the untreated specimens were set as baseline and showed the initial phase composition prior to mechanical and hydrothermal accelerated aging (Figure 6, position (1)A).

The proportion of the monoclinic phase decreased after mechanical processing (grinding) and entirely disappeared after chewing and polishing (Figure 6, position (1)B).

Thermocycling predominantly caused a slight increase in the monoclinic phase fractions and made it difficult to distinguish between the different phases (Figure 6, positions (1)A, (1)C, and (1)D).

Chewing completely transformed the monoclinic fraction into a tetragonal phase (Figure 6, position (2)E).

With increasing yttria content, the proportion of the tetragonal phase t″ increased, while the proportions of the tetragonal phase t and the monoclinic phase decreased accordingly.

Differences between the Y-TZPs from the two manufacturers were within the limits of measurement accuracy. The values identified were comparable with those reported by Keuper et al. [14].

## 4. Discussion

In the current study, the monoclinic phase fraction transformed entirely to a tetragonal phase under mechanical stress (cf. Figure 6) [40]. These findings are in contrast to other studies where no monoclinic fraction was identified at all [29] or where the monoclinic phase fraction increased by thermal [3,14,15,41] or mechanical [2,16,17] stress (which may be supported by water [3,14,15]).

From a thermodynamic point of view, a phase transformation from a stable phase into a high-temperature (pressure) phase (tetragonal) could only be induced by a sufficient local input of energy.

There are several publications [42,43,44] that report a transformation of the monoclinic phase into the tetragonal phase of “pure zirconia” with the combination of moderately increased pressure and temperature (e.g., 2.2 GPa/600 °C [42]), which could be even lower due to the yttria stabilization.

In the current study, the phase boundary between the monoclinic and tetragonal phases must have been passed by a high energy input (grinding and polishing process), which can be explained by three combinable theories:

As erosion and compression of the materials was visible, the mechanical load induced a critical stress state, which exceeded at least the strength.

Since partially stabilized zirconia is a poor heat conductor [45,46], high local temperatures are possible.

The tetragonal phase t features a higher density than the monoclinic phase. Thus, the multiphase system might avoid the appearance of pressure by compression (i.e., transformation into one of the tetragonal phases).

The formula of Garvie and Nicholson [38] in combination with the correction by Toraya [39] only applies to a homogeneous powder mixture with a homogeneously distributed monoclinic phase (no preferred orientation). In order not to increase the half-width, the phases must have crystallized sufficiently well and not have changed due to the treatments applied [36].

Furthermore, it must be taken into account that the analyzed sample volume depends on the measurement setup (measurement geometry, wavelength used, and intensity of the primary beam) employed [41].

**Figure 7:**
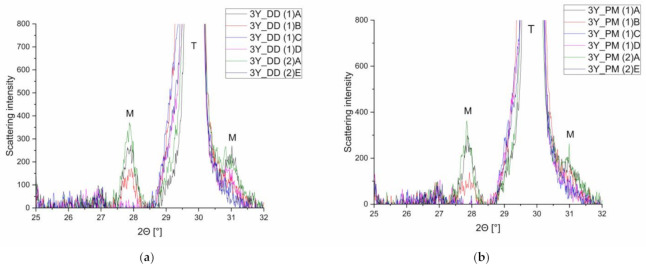
Comparison of the observed curves at the six different measurement positions for two 3y-TZP samples at around 30°. Their monoclinic (m) and tetragonal (t) reflexes are indicated. (**a**) 3y_DD, (**b**) 3y_PM.

Generally, the number of XRD reflexes increases with decreasing symmetry of the phases. Further reflexes occur, although they are in some cases accompanied by low intensity. Monoclinic crystal systems have lower symmetry, and, therefore, the number of reflexes is high, and the intensity of each reflex is low. This method is particularly prone to errors in systems with small monoclinic components since the monoclinic reflections hardly stick out of the noise (cf. Figure 7).

Thus, the proportions determined by this procedure should not be regarded as absolute numbers but rather seen as describing the progress of phase transformation, thereby allowing comparisons with other studies.

While the absolute fraction of the monoclinic phase is up to three times higher with the Rietveld refinement, the proportions identified for the changes in the fraction of the monoclinic phase coinciding with a continuous increase in the yttria fraction are similar. However, halving of the proportion of the monoclinic phase from A (reference) to processing step B (grinding) is not discernible with the formula of Garvie and Nicholson [38,39].

As the diffractograms (Figure 7) clearly show the transformation of the monoclinic phase (2θ = 27.5–28.4°) in two stages, it is obvious that the Rietveld refinement is far better than the method of Garvie and Nicholson [38].

With regard to the tetragonal phase t′, it should be noted that it is very similar to the tetragonal phase t and that portions of t can be very easily attributed to the tetragonal phase t′ (“tetragonality” t: 1.0159; t′: 1.0149; t″: 1.0033 [36]). Since removing the tetragonal phase t′ affects the refinement only insignificantly, the distinction between the tetragonal phases t′ and t is doubtful. On the other hand, there are several papers [23,24,25] that describe the occurrence of the tetragonal phase t′ in a non-dental context.

## 5. Conclusions

Despite the limitations of the present study, the null hypothesis cannot be confirmed. The following conclusions were drawn:

(1) X-ray diffraction (XRD) is a suitable method for analyzing the phase composition of yttria-stabilized zirconia.
(2) Mechanical processing partly (grinding) or completely (polishing and chewing) transforms the monoclinic phase into the tetragonal phase in all specimens investigated. Low-temperature degradation was not observed in the current study.
(3) The composition of the tetragonal phase is dependent on the yttria content. In specimens with a content of 3 mol% yttria, the tetragonal phase t was preferred, while in specimens with a content of 5 mol% yttria, the tetragonal phase t″ was preferred.
(4) The Rietveld refinement is far more accurate for the evaluation of XRD data than the Garvie-Nicholson method [38,39] (DIN EN ISO 13,356 [37]).

Due to the limited number of specimens (two to three per yttria content) and measurements per position, this study can only suggest future approaches. In order to achieve reproducible and comparable test results, the influences (of sampling) and measurement settings (X-ray tube, detector, and measurement count) should be investigated more extensively. The current study could not verify whether a failure-controlled (cause of damage: local stress peaks, consequence of phase transformation) or a transformation-controlled (cause: Phase transformation, consequence of micro crack formation) failure pattern occurred.

## Figures and Tables

**Table 1:** List of the used sample plates from the manufactures pritidenta GmbH (Germany, PM) and Dental Direct GmbH (Germany, DD). HF means “high-strength”, a product of DD, according to its own description with higher strength and lower translucency than the other three yttria-stabilized tetragonal zirconia polycrystalline (3y-TZP) product 3y_DD.

Abbreviation	Product	Manufacturer	Yttria Content (mol%)	Flexural Strength (MPa) ^1^
3y_PM	Priti multi translucent	PM	3	>1150
3y_HF	DD Bio ZW iso	DD	3	>1300
3y_DD	DD Bio ZX^2^	DD	3	>1100
4y_PM	Priti multi extra translucent	PM	4	>1150
4y_DD	DD cube ONE	DD	4	>1250
5y_PM	Priti multi high translucent	PM	5	>650
5y_DD	DD cubeX^2^	DD	5	>750

^1^ According to the manufacturer.

**Table 2:** Description of the different types of treatment: (Sample plate: (1)—without thermocycling (TC), (2)—with thermocycling (TC) and chewing simulation). Number: Abbreviations of surface treatments.

Abbreviationof Treatment	Treatment	Number of Cycles
(1)A	Untreated	-
(1)B	Grinding (red ring diamond)	-
(1)C	Grinding and polishing	-
(1)D	Polishing (polishing kit)	-
(2)A	Thermocycling 5–55 °C	10,000 (2 min each)
(2)E	1. Grinding and polishing	-
	2. Chewing simulation	1.2 × 10^6^ (50 N; 1 Hz; enstatite antagonist)
	3. Thermocycling 5–55 °C	10,000 (2 min each)

## Data Availability

The authors state that no external data were used.

## Appendix A

List of the statistically significantly different pairwise comparisons:

- *z*-score:

The standard score, known also as *z*-score, describes the number of standard deviations by which the value of the observed measurement position differs positively or negatively from the mean value of all measurement positions.

- *p*-value:

The *p*-value shows the probability of measuring a value that is as extreme as the measurements that were actually observed, assuming that the null hypothesis is correct and the actual hypothesis is false.

A very small *p*-value therefore means that such a measurement would be extremely unlikely under the null hypothesis.

For these analyses, we chose a significance level of alpha = 0.05.

- *r*-value:

The *r*-value indicates the standardized effect size according to Cohen [32]. It ranges from 0 to 1 and is estimated as follows: *r* = 0.1 to 0.3 means a small effect; 0.3 to 0.5 indicates an intermediate effect; 0.5 and higher indicates a strong effect.

**Table A1:** Ceramic 3y_DD (χ^2^(4) = 110.00; *p* < 0.000); N = 125.

Measurement Point	*Z*	*p*	*r*
AB	−2.440	=0.147	-
AC	5.254	<0.000 **	0.47
AD	2.799	=0.051	-
AE	6.585	<0.000 **	0.59
BC	7.694	<0.000 **	0.69
BD	5.239	<0.000 **	0.47
BE	9.025	<0.000 **	0.81
CD	−2.455	=0.141	-
CE	1.331	>1.000	-
DE	3.787	=0.002 *	0.34

* *p* < 0.05; ** *p* < 0.001.

**Table A2:** Ceramic 3y_PM (χ^2^(4) = 99.26; *p* < 0.000); N = 125.

Measurement Point	*Z*	*p*	*r*
AB	−2.459	=0.139	-
AC	4.481	<0.000 **	0.40
AD	4.007	=0.001 *	0.36
AE	6.053	<0.000 **	0.54
BC	6.941	<0.000 **	0.62
BD	6.466	<0.000 **	0.58
BE	8.512	<0.000 **	0.76
CD	−0.474	>1.000	-
CE	1.571	>1.000	-
DE	2.046	=0.408	-

* *p* < 0.05; ** *p* < 0.001.

**Table A3:** Ceramic 3y_HF (χ^2^(4) = 102.02; *p* < 0.000); N = 125.

Measurement Point	*Z*	*p*	*r*
AE	0.015	=0.147	-
AC	3.377	=0.007 *	0.30
AD	5.652	<0.000 **	0.51
AE	5.609	<0.000 **	0.50
BC	5.816	<0.000 **	0.52
BD	8.092	<0.000 **	0.72
BE	8.094	<0.000 **	0.72
CD	2.276	=0.229	-
CE	2.233	=0.256	-
DE	−0.043	>1.000	-

* *p* < 0.05; ** *p* < 0.001.

**Table A4:** Ceramic 4y_DD (χ^2^(4) = 108.09; *p* < 0.000); N = 125.

Measurement Point	*Z*	*p*	*r*
AB	−2.440	=0.147	-
AC	3.111	=0.019 *	0.28
AD	6.695	<0.000 **	0.56
AE	4.833	<0.000 **	0.43
BC	5.551	<0.000 **	0.50
BD	9.134	<0.000 **	0.82
BE	7.272	<0.000 **	0.65
CD	3.584	=0.003 *	0.32
CE	1.721	=0.852	-
DE	−1.862	=0.626	-

* *p* < 0.05; ** *p* < 0.001.

**Table A5:** Ceramic 4y_PM (χ^2^(4) = 104.5; *p* < 0.000); N = 125

Measurement Point	*Z*	*p*	*r*
AB	−2.440	=0.147	-
AC	4.110	<0.000 **	0.37
AD	3.896	=0.001 *	0.35
AE	6.632	<0.000 **	0.59
BC	6.550	<0.000 **	0.59
BD	6.336	<0.000 **	0.57
BE	9.072	<0.000 **	0.81
CD	−0.215	>1.000	-
CE	2.522	=0.117	-
DE	2.736	=0.062	-

* *p* < 0.05; ** *p* < 0.001.

**Table A6:** Ceramic 5y_DD (χ^2^(4) = 102.02; *p* < 0.000); N = 125.

Measurement Point	*Z*	*p*	*r*
AB	−2.440	=0.147	-
AC	3.466	=0.001 *	0.31
AD	5.141	<0.000 **	0.46
AE	6.031	<0.000 **	0.54
BC	5.906	<0.000 **	0.53
BD	7.581	<0.000 **	0.68
BE	8.471	<0.000 **	0.76
CD	1.675	=0.940	-
CE	2.565	=0.103	-
DE	0.890	>1.000	-

* *p* < 0.05; ** *p* < 0.001.

**Table A7:** Ceramic 5y_PM (χ^2^(4) = 99.73; *p* < 0.000); N = 125.

Measurement Point	*Z*	*p*	*r*
AB	−2.440	=0.147	-
AC	3.724	=0.002 *	0.33
AD	5.805	<0.000 **	0.52
AE	5.110	<0.000 **	0.46
BC	6.164	<0.000 **	0.55
BD	8.244	<0.000 **	0.74
BE	7.550	<0.000 **	0.68
CD	2.081	=0.375	-
CE	1.386	>1.000	-
DE	−0.695	>1.000	-

* *p* < 0.05; ** *p* < 0.001.
